# Miscellany

**Published:** 1884-01

**Authors:** 


### Miscellany.

### THE HEARING OF SCHOOL CHILDREN.

In an article on the results of an examination of the ear and the hearing of 5,905 school children, in the *Archives of Otology*, Vol. XI, No. 1, Dr. Weil makes some very sensible remarks. He considers, for instance, that every inattentive child should have its ears examined—so convinced is he of the fact that children who are simply hard of hearing are often misjudged and considered inattentive. Of course, it would be much better, he says rightly, if every school child underwent such an examination once or twice every year. It is not necessary that such an examination should be made by a medical man, since the teachers could do it, but of course not so well as the physician. It does not require much time or trouble, certainly not more than one hour for each class. The test could be made in the school room itself if there be no other room convenient. The teacher could place the pupil in one corner of the room, then retire to the other himself, and test each ear separately by whispering. He should cause the words and sentences used to be repeated by the pupil, and could thus easily find out which of them are hard of hearing. This would have the further advantage of calling the attention of parents to the condition of their children, thus preventing injustice from being done to them, and making them profit by early treatment. The author believes that, in the great majority of cases, the children whom he examined could be much benefitted by proper treatment, and many of them could be entirely relieved in a few minutes. Probably the great majority, he says, will never be submitted to proper treatment, or at least not till after some years, when the disease will have caused changes which can then be but little benefitted. The author thinks that many of the children will be neglected by their parents, even after they become informed of their condition, simply on account of the cost of treatment; and he, therefore, recommends the appointment of a proper surgeon to be responsible for the health of schools, and whose duty it would be to examine the ears of every child whom the teachers find inattentive, and, when necessary, to give the proper advice.—*British Medical Journal.*

---

ENTHUSIASM is one of the most powerful engines of success. When you do a thing, do it with a vim. Do it with your might. Put your whole soul into it. Stamp it with your own personality. Be active, be energetic, be enthusiastic and faithful, and you will accomplish your object. Truly has Emerson said: "Nothing great was ever achieved without enthusiasm."

